# Tuning nanoporous anodic alumina distributed-Bragg reflectors with the number of anodization cycles and the anodization temperature

**DOI:** 10.1186/1556-276X-9-416

**Published:** 2014-08-21

**Authors:** Josep Ferré-Borrull, Mohammad Mahbubur Rahman, Josep Pallarès, Lluís F Marsal

**Affiliations:** 1 Department of Electronic, Electric and Automatics Engineering, Universitat Rovira i Virgili, Avda. Països Catalans 26, Tarragona 43007, Spain

**Keywords:** Nanoporous anodic alumina, Distributed-Bragg reflectors, Photonic properties tuning, Anodization temperature, Pore widening, Cyclic voltage anodization

## Abstract

The influence of the anodization temperature and of the number of applied voltage cycles on the photonic properties of nanoporous anodic alumina-based distributed-Bragg reflectors obtained by cyclic voltage anodization is analyzed. Furthermore, the possibility of tuning the stop band central wavelength with a pore-widening treatment after anodization and its combined effect with temperature has been studied by means of scanning electron microscopy and spectroscopic transmittance measurements. The spectra for samples measured right after anodization show irregular stop bands, which become better defined with the pore widening process. The results show that with 50 applied voltage cycles, stop bands are obtained and that increasing the number of cycles contributes to enhancing the photonic stop bands (specially for the case of the as-produced samples) but at the expense of increased scattering losses. The anodization temperature is a crucial factor in the tuning of the photonic stop bands, with a linear rate of 42 nm/°C. The pore widening permits further tuning to reach stop bands with central wavelengths as low as 500 nm. Furthermore, the results also show that applying different anodization temperatures does not have a great influence in the pore-widening rate or in the photonic stop band width.

## Background

Nanoporous anodic alumina (NAA) is a material of great interest in nanotechnology because of its cost-effective and easily up-scalable production techniques [1-3] and also because of its vast field of applications [4-8]. This material consists of an array of cylindrical pores in an aluminum oxide matrix obtained by electrochemical anodization of aluminum. In the appropriate fabrication conditions, the pores self-arrange in a triangular lattice with domains containing several hundreds of pores [9]. This pore arrangement is usually obtained with three kinds of acid electrolytes (oxalic, phosphoric, or sulfuric) and in two different regimes, known as hard and mild anodization [10].

The photonic properties of NAA make this material specially interesting in optical applications such as biosensing [11-14] In previous works, the authors described the existence of photonic stop bands for light propagating inside the material [15] in a direction perpendicular to the pore axes, and also described a method to obtain distributed-Bragg reflectors (DBRs) [16] based on NAA. DBRs are dielectric multilayer structures [17-20] with a periodic variation of the refractive index in the direction perpendicular to the surface. This gives rise to photonic stop bands for light incident in a direction parallel to the pore axes. The central wavelength of such stop bands depends on the effective refractive index and on the optical thickness of each of the cycles, while the width of the bands is directly related with the contrast of the refractive index variations. Ideal photonic stop bands are achieved for infinite periodic structures [21,22]. However, DBR structures are finite and consequently, the characteristics of the photonic stop band depend on the number of cycles they contain.

NAA-based DBR can be achieved by taking advantage of the fact that a wet etching applied after the anodization to enlarge the pore diameter (pore-widening step) has a different rate depending on the used anodization voltage [23]. Thus, by combining a cyclic anodization voltage with a subsequent pore-widening step, tunable in-depth modulation of the pore diameter and effective refractive index variations are obtained. Other authors have reported on the fabrication of DBR structures by applying a cyclic anodization voltage [19,20,24] although they did not stress the importance of the pore-widening step in order to obtain the photonic stop bands.

Temperature is also a key factor in the fabrication of NAA structures [25,26], as it is directly influencing the reaction speed. By lowering adequately the temperature, an increase in anodization voltage is possible so that hard-anodization NAA can be obtained without the need of an initial protective layer [25]. The color of the NAA can also be influenced by temperature [26].

In this work, we study the influence of the number of cycles and of the anodization temperature on the optical properties of NAA-based DBR. We also study how the pore-widening step (necessary to obtain the well-defined photonic stop bands) can be combined with these parameters in order to adjust the stop band position of the fabricated structures.

## Methods

For the synthesis of NAA-based DBR, we have used high-purity Al substrates (99.99%) of 500-μm thickness from Sigma-Aldrich (St. Louis, MO, USA). A pretreatment is required to meliorate the physical properties of the commercial Al substrate: first, the Al substrates were rinsed in deionized water, then cleaned with ethanol and rinsed in deionized water again, then dried with N_2_ and stored in a dry environment. Then, the surface roughness was reduced by an electropolishing process performed at room temperature and at 20 V for 4 min in a 1:4 *v*/*v* mixture of perchloric acid and ethanol. The sense of the stirrer was switched every 1 min. After electropolishing, the samples were cleaned in water. A first anodization was performed on the electropolished Al surface using 0.3 M oxalic acid (H_2_C_2_O_4_) solution at a temperature of 7°C. The anodization process was carried out in a PVC cell cooled by a circulating system (Thermo Scientific, Waltham, MA, USA) with continuous stirring, which ensured a stabilized temperature within an accuracy of less than 0.5°C. The working surface area of the samples was 1.4 cm^2^. A Pt grid was used as a cathode, and the distance between the two electrodes was about 2 cm. The electrochemical process was controlled by a lab-view program that saved the data of current and voltage and the amount of charge flown through the system every 200 ms. The process was carried out at a constant voltage (V) of 40 V for 20 h. The resulting nanostructure after this first anodization step is a thin film of alumina with disordered pores at the top but self-ordered pores at the bottom. This alumina film was dissolved by wet chemical etching at 70°C in a solution of chromic and phosphoric acids (0.4 M H_3_PO_4_ and 0.2 M H_3_CrO_4_), stirred at 300 rpm for 4 h. A number of samples were prepared in order to examine the effect of the applied number of cycles (*N*_C_) and of the anodization temperature (*T*_anod_).

In order to examine the effect of the number of cycles, two types of samples having different *N*_C_ were fabricated. A detail of the applied anodization voltage to one of the samples is shown in Additional file 1: Figure S1 where Figure S1(a) in Additional file 1 represents the voltage profile of entire anodization process with 50 cycles, while Figure S1(b) in Additional file 1 represents the voltage profile of one cycle. The anodization process started at 20 V and it lasted until a charge of 2 C flowed through the system. In this way, a self-ordered layer of vertical pores was obtained. To obtain the DBR structure, after this anodization at 20 V, the cyclic anodization process started immediately. Each cycle consisted of three phases: (I) a linear increasing ramp from 20 to 50 V, at a rate of 0.5 V/s, (II) an interval at 50 V for certain time duration to flow a given charge *Q*_0_ through the system, and (III) a subsequent linear decreasing ramp from 50 to 20 V at 0.1 V/s. The increasing and decreasing ramps were chosen as the fastest possible ramps in order to maintain the continuity of the anodization process. After the cyclic anodization steps finished, a final anodization voltage of 20 V was applied until 2 C of charge flowed through the system. After the anodization, a wet etching to increase pore radius (pore-widening step) was performed with 5 wt.% phosphoric acid (H_3_PO_4_) at 35°C. This pore widening was applied for different times, *t*_PW_. Samples with *N*_C_ = 50 and *N*_C_ = 150 cycles were obtained, with a *Q*_0_ = 0.5 C. On the other hand, samples with *Q*_0_ = 0 C were produced at four different anodization temperatures: *T*_anod_ = 8, 9, 10, and 11°C.

## Results and discussion

**Figure 1:**
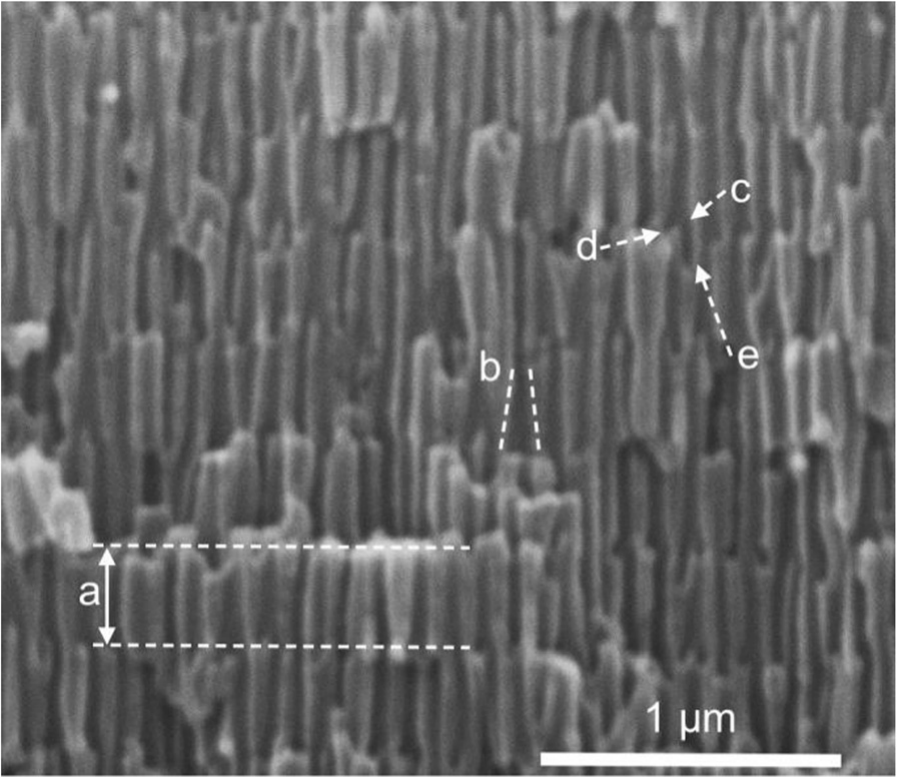
**SEM cross-section picture of NAA-based DBR sample obtained with Q**_**0**_ **= 0.5 C, 50 cycles, and *T***_**anod**_ **= 9°C.** ‘a’ interfaces limiting one cycle, ‘b’ pore with conical shape, ‘c’ beginning of a pore branching corresponding to a decreasing anodization voltage, ‘d’ frustrated branch as the voltage increases again, and ‘e’ surviving pore growing in the subsequent cycle.

Figure 1 shows the scanning electron microscpy (SEM) cross-section image of the sample produced with *Q*_0_ = 0.5 C, *N*_C_ = 50, and *T*_anod_ = 9°C. The picture shows the in-depth pore modulation caused by the cyclic voltage. Seven cycles can be recognized, separated by interfaces consisting of abrupt changes in the pore diameter and morphology. Within one cycle (indicated by a letter ‘a’ in the picture), the pores show mainly conical shapes (‘b’), with a smaller diameter in the upper part of the cycle. At the lower part of the cycle, the pores start to branch (‘c’), although at some point, the branching is frustrated (‘d’) and only one of the branches continues as a new pore in the next cycle (‘e’). These facts indicate that the visible interfaces between the pores correspond to the lower voltage in the cycle, since the pore branching begins to occur with the reduction of the voltage. However, the branching is frustrated by the immediate increase of the voltage as it reaches the 20-V value with the consequent single-pore development further into the next cycle.

**Figure 2:**
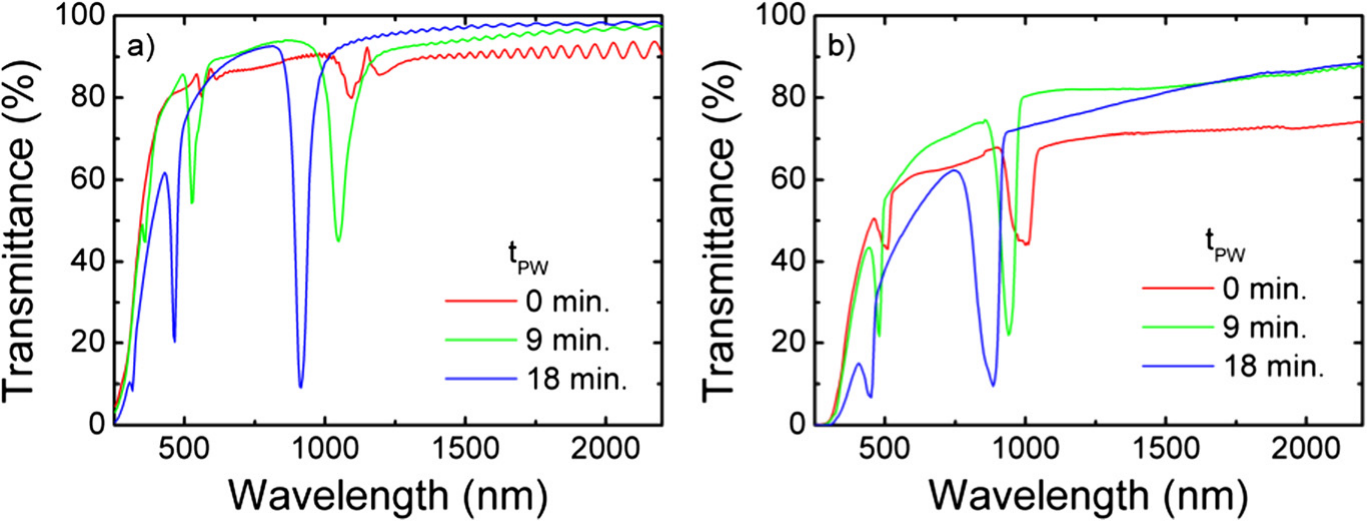
**Comparison of the spectra of samples obtained with N**_**C**_ **= 50 cycles (a) and N**_**C**_ **= 150 cycles (b).**

The effect of applying different number of cycles to obtain the NAA-based DBR can be deduced from the transmittance spectra shown in Figure 2. The plots show the spectra for a sample produced with *N*_C_ = 50 and *T*_anod_ = 9°C (a) and a sample with *N*_C_ = 150 and *T*_anod_ = 7°C (b) after different pore-widening times (*t*_PW_ = 0, 9, and 18 min). All the spectra show two stop bands (spectral ranges with reduced transmittance): the first-order stop band at higher wavelengths and also a second-order stop band at half of the wavelength of the first one. It is interesting to remark that the spectra for the as-produced samples (*t*_PW_ = 0 min) show irregular stop bands, especially for the sample with *N*_C_ = 50 that shows even a local transmittance maximum at 1,152 nm. This is usual in NAA-based DBR obtained with a cyclic voltage [16] and is explained by the fact that porosity depends weakly on anodization voltage, and in consequence, voltage variations create morphology changes in the pores as they grow but small changes in porosity. Nevertheless, it is worth to note that the stop band for the as-produced 150-cycle sample shows a more pronounced decrease in the transmittance within the stop band. Thus, even though the refractive index contrast is small, a higher number of cycles and the corresponding higher number of cycle interfaces contribute to enhance the photonic stop band properties. The spectra for the samples after some pore widening show clearly defined stop bands with decreasing central wavelength for increasing pore widening time. Additional file 1: Table S1 summarizes the values of central wavelength and stop band width of the spectra. By comparing the ranges in the spectra not corresponding to a stop band, it can be concluded that the transmittance for *N*_C_ = 150 is lower than for *N*_C_ = 50. This difference can be attributed to scattering losses caused by the irregular interfaces between each cycle. Finally, there is a clear difference between the central wavelength of the stop bands, which is lower for the sample produced at the lower temperature, *N*_C_ = 150 and *T*_anod_ = 7°C.

**Figure 3:**
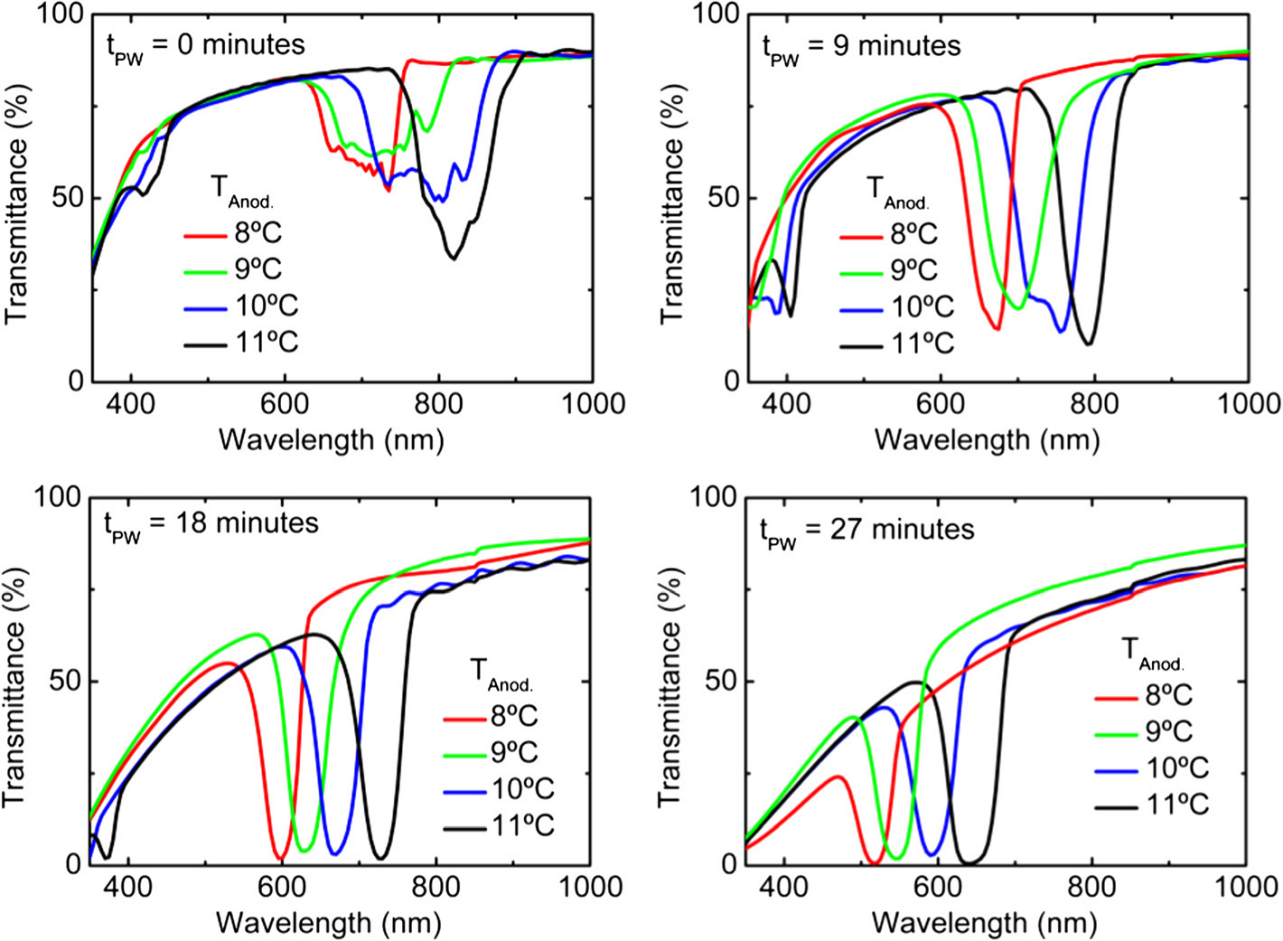
Comparison of the spectra of samples obtained at different anodization temperatures and after different pore-widening times.

In order to evaluate more precisely this dependence of the stop band central wavelength with the temperature, Figure 3 shows the transmittance spectra for samples produced with temperatures *T*_anod_ = 8, 9, 10, and 11°C and after different times of pore widening, *t*_PW_ = 0, 9, 18, and 27 min. The spectra show similar trends as the observed in Figure 2: for the as-produced samples, the spectra show truncated stop bands that become better defined with the pore-widening process. At the same time, the pore widening causes a decrease in the central wavelength as it decreases the overall effective refractive index of each cycle in the DBR. Additional file 1: Table S2 reports the values of stop band central wavelength and stop band width for the spectra. The spectra in Figure 3 show that the main influence of the anodization temperature is in the stop band central wavelength, while other features such as the depth of the stop band transmittance minimum or the difference in shape observed for the as-produced samples are less influenced by *T*_anod_.

**Figure 4:**
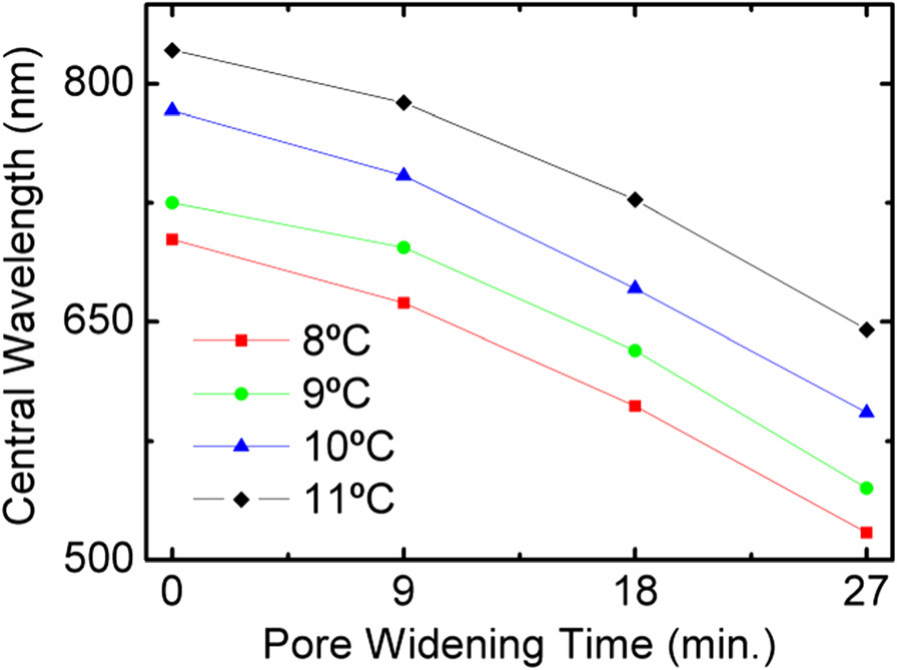
Evolution of central wavelength of the first stop band as function of pore-widening time for different anodization temperatures.

The dependence of the central wavelength with the anodization temperature is summarized in Figure 4, where the different central wavelengths of the first-order stop band are plotted as a function of the pore-widening time. The data in Figure 4 demonstrate that by a precise control of the temperature and of the pore-widening time, the stop band central wavelength can be modulated between 500 and 820 nm. The curves for the different temperatures show the same behavior, what indicates that carrying the anodization at a different temperature does not influence the pore-widening rate in the subsequent pore-widening process. It is also important to mention that the intervals between the curves in Figure 4 are constant, what indicates that the shift of the central wavelength with the temperature is uniform with an estimated average value of 42.5 nm/°C (see Additional file 1: Figure S2). Table 1 shows the average stop band width for the different pore-widening times and the corresponding standard deviation. This average and standard deviation for a given pore-widening time are obtained considering all the values for the different temperatures. The results show that there is a small dispersion in stop band width for the different temperatures. Since the stop band width depends basically on the refractive index contrast that can be achieved within a cycle, it can be concluded that the anodization temperature has a small influence in the refractive index contrast.

**Table 1:** Average stop band width and corresponding standard deviation as a function of the pore-widening time

**Pore-widening time (min)**	**Average stop band width (nm)**	**Stop band width standard deviation (nm)**
0	103	22
9	68	14
18	50	5
27	46	6

The average and standard deviation have been obtained for all the samples with a given pore-widening time and different temperatures. The small value of the standard deviation as compared with the average stop band width indicates that the temperature has a small influence in the refractive index contrast obtained with the cyclic voltage anodization.

## Conclusions

In this work, we analyzed the influence of the anodization temperature and of the number of applied voltage cycles on the photonic properties of NAA-based DBRs obtained by cyclic voltage anodization. In previous works, it was shown that DBR structures with stop bands can be obtained by the application of an anodization based in the repetition of voltage cycles between 20 and 50 V in 0.3 M oxalic acid. It was also shown that the application of a pore-widening step after anodization is crucial in order to obtain well-defined stop bands with low transmittance and high reflectance. In this work, these nanoporous structures have been obtained in the range of temperatures between 8°C and 11°C, for 50 and 150 applied voltage cycles and pore-widening times up to 27 min. The effect of these parameters on the morphologic and photonic properties of the nanostructures has been studied by means of SEM and spectroscopic transmittance measurements.

The results show that 50 applied voltage cycles are enough to produce stop bands and that increasing the number of cycles has two opposite effects: on one hand, an enhancement of the photonic stop bands is observed, in particular specially for the case of the as-produced samples, which is much better defined for samples with higher number of cycles. On the other hand, scattering losses are observed in the spectra caused by the irregular interfaces between cycles observed in the SEM images. Such losses increase with increasing number cycles and the corresponding interfaces.

Increasing the anodization temperature produces a remarkable shift of the photonic stop band central wavelength, with a linear rate of 42.5 nm/°C. On the other hand, a change in anodization temperature does not influence noticeably the obtained stop band widths or the rate of the subsequent pore widening. These three facts suggest that anodization temperature has a strong effect on the pore growth rate during anodization, but a small influence on the pore diameter or morphology. With this, it is also put into evidence that a precise control and stabilization of the temperature along the whole fabrication process is crucial to ensure accuracy in the tuning of the photonic stop bands.

## Abbreviations

DBR: distributed-Bragg reflector; NAA: nanoporous anodic alumina; *N*_C_: number of anodization voltage cycles; *T*_anod_: anodization temperature; *t*_PW_: pore-widening time; SEM: scanning electron microscopy

## Competing interests

All the authors declare that they have no competing interests.

## Authors' contributions

MMR, LFM, and JFB designed the experiment and analyzed and discussed the results. MMR fabricated the NAA-based DBR and performed the optical characterization. All authors redacted and revised the manuscript. All authors read and approved the final manuscript.

## Supplementary Material

Additional file 1 **Applied cyclic anodization voltage, linear fits of the evolution of the stop band central wavelength, and central wavelength and width of the first-order stop band.** Example of the applied cyclic anodization voltage, linear fits of the evolution of the stop band central wavelength with the temperature for the different applied pore widening times, and central wavelength and width of the first-order stop band for the samples obtained with different number of cycles and different anodization temperatures. Click here for file
