# Supplementary material for: Tuning nanoporous anodic alumina distributed-Bragg reflectors with the number of anodization cycles and the anodization temperature
Source: Nanoscale Res Lett. 2014 Aug 21;9(1):416. doi: 10.1186/1556-276X-9-416 (PMC4147935; doi:10.1186/1556-276X-9-416)
Supplement: Additional file 1 — Applied cyclic anodization voltage, linear fits of the evolution of the stop band central wavelength, and central wavelength and width of the first-order stop band. Example of the applied cyclic anodization voltage, linear fits of the evolution of the stop band central wavelength with the temperature for the different applied pore widening times, and central wavelength and width of the first-order stop band for the samples obtained with different number of cycles and different anodization temperatures. [file 1556-276X-9-416-S1.doc]

Additional file 1

**Tuning Nanoporous Anodic Alumina Distributed Bragg Reflectors with the Number of Anodization Cycles and the Anodization Temperature**

**
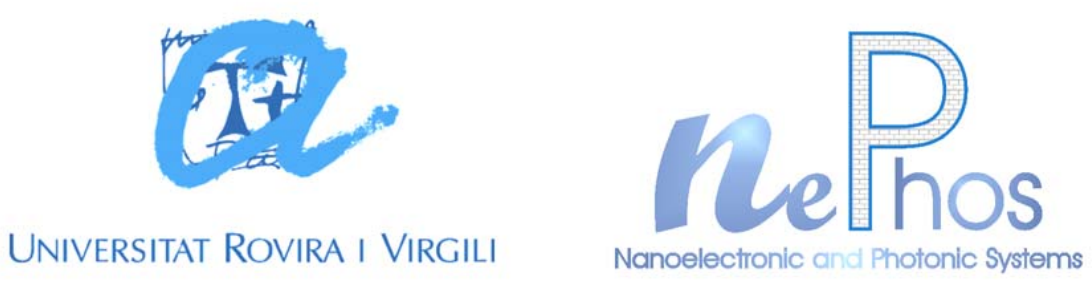
**

Josep Ferré-Borrull, Mohammad Mahbubur Rahman, Josep Pallares, and Lluís F. Marsal

Nano-electronic and Photonic Systems (NePhoS)

Departament d’Enginyeria Electrònica, Elèctrica i Automàtica

Universitat Rovira i Virgili.

Avda. Països Catalans 26, 43007 Tarragona, Spain.

**Figure S1: Example of the applied cyclic anodization voltage.** (a) Complete anodization process with N_C_ = 50 cycles used to produce the sample depicted in Figure 1 in the paper. (b) Detail of one of the cycles with (i) the increasing voltage ramp (0.5 V/s), (ii) the constant voltage phase that lasts until a charge Q_0_ flows through the system and (iii) the decreasing voltage ramp (0.1 V/s).


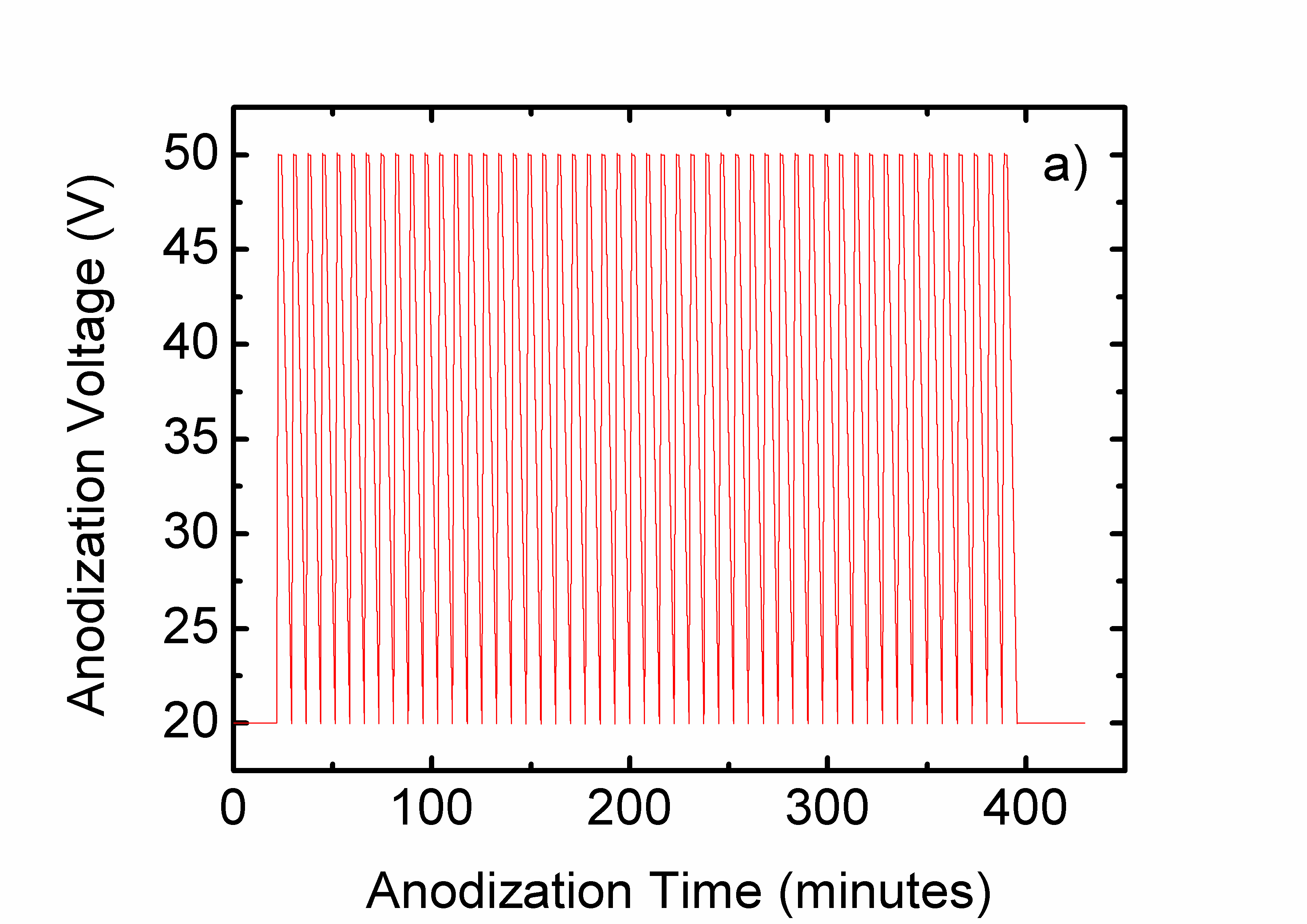

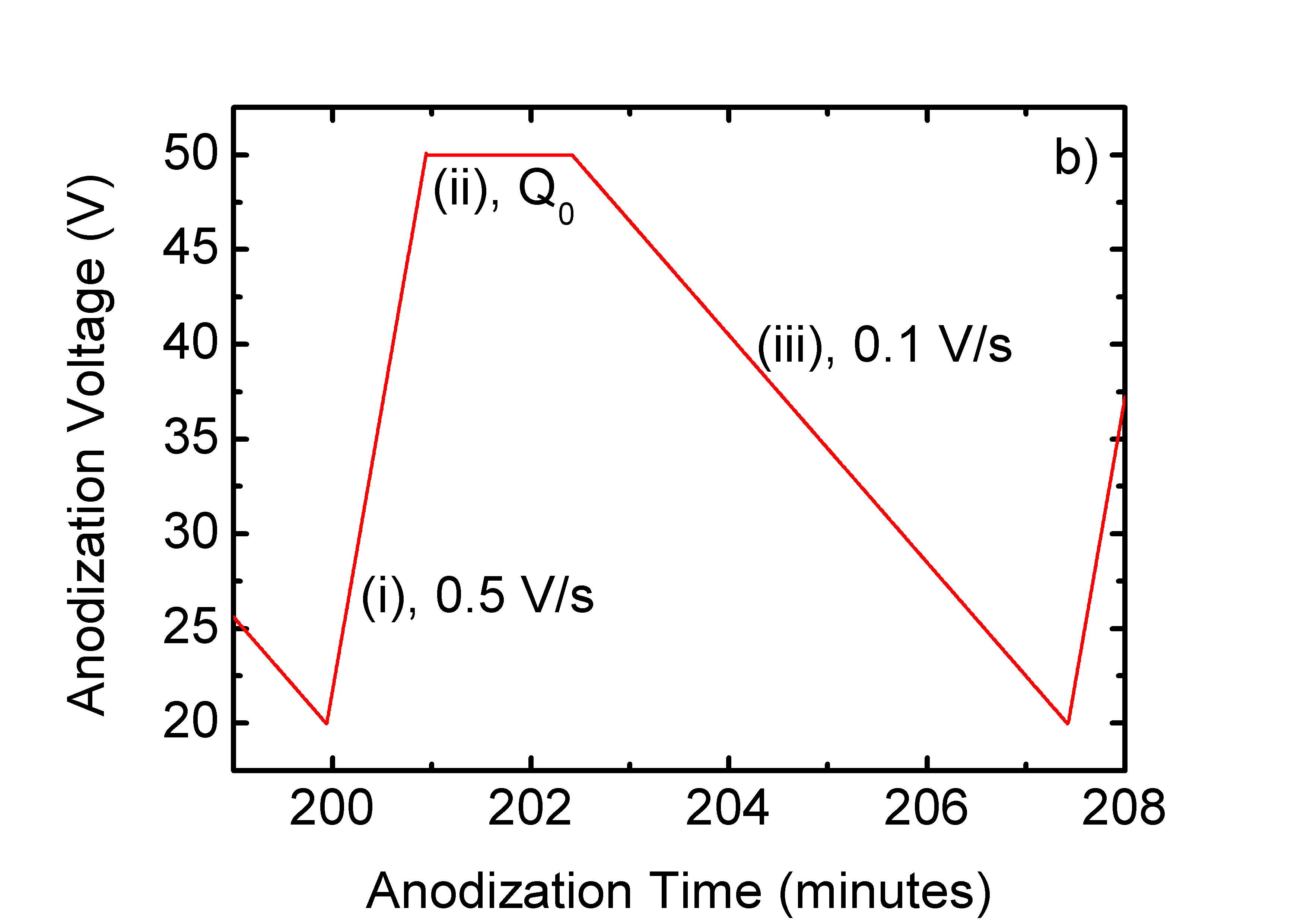


**Figure S2: Linear fits of the evolution of the stop band central wavelength with the temperature for the different applied pore widening times.** Red squares (◼): as-produced samples, green circles (⚫): samples after 9 minutes of pore widening, blue triangles (⯅): samples after 18 minutes of pore widening, black rhombi (⯁): samples after 27 minutes of pore widening.

**
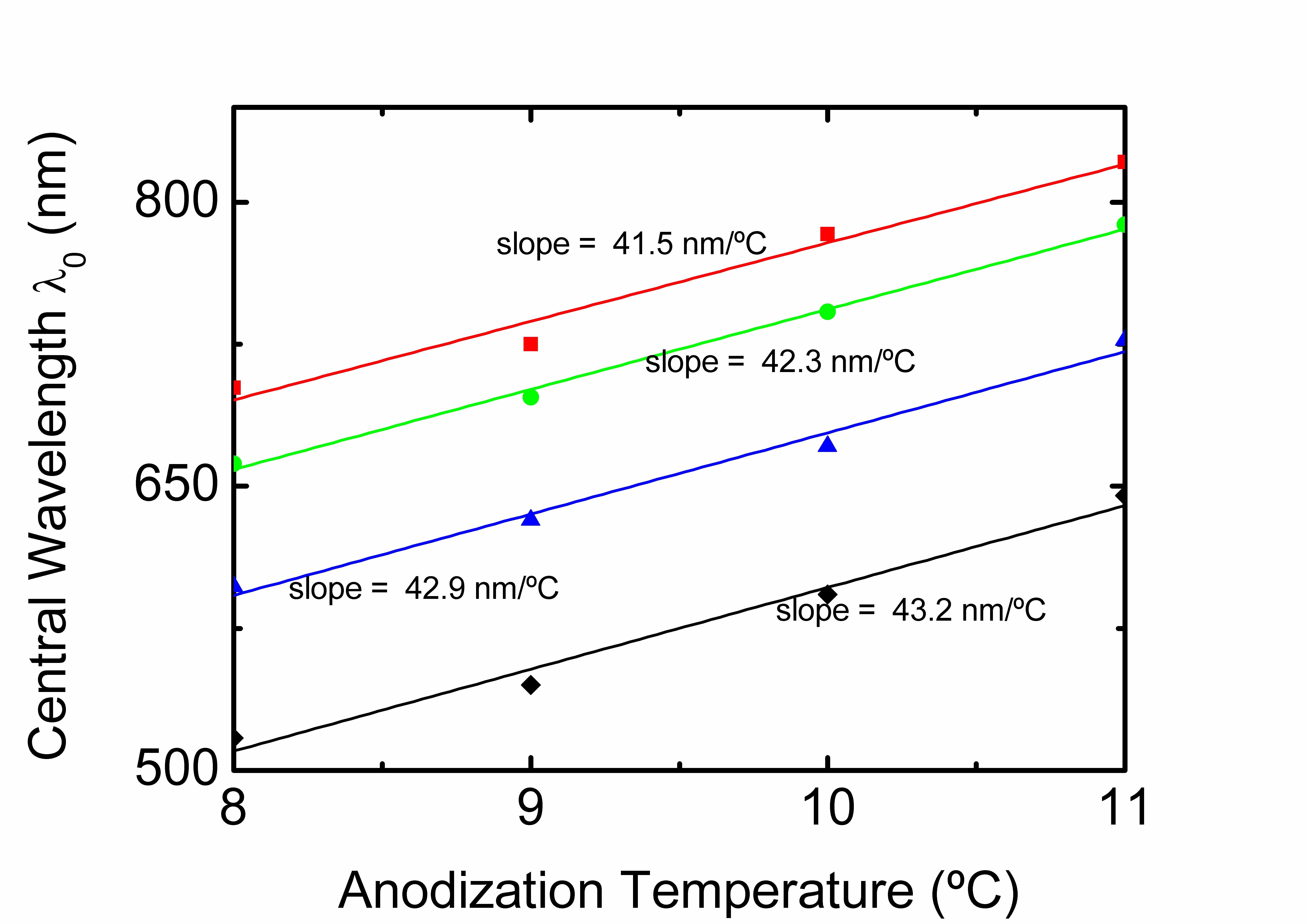
**

**Table S1. Central wavelength and width of the first-order stop band for the samples obtained with different number of cycles**.

| Number of cycles | Pore Widening Time (minutes) | Central Wavelength (nm) | Stop Band Width (nm) |
| --- | --- | --- | --- |
| 50 | 0 | 1099 | 48 |
|  | 9 | 1053 | 79 |
|  | 18 | 916 | 53 |
| 150 | 0 | 984 | 58 |
|  | 9 | 937 | 52 |
|  | 18 | 866 | 77 |

**Table S2. Central wavelength and width of the first-order stop band for the samples obtained with different anodization temperatures**.

| Anodization Temperature (ºC) | Pore Widening Time (minutes) | Central Wavelength (nm) | Stop Band Width (nm) |
| --- | --- | --- | --- |
| 8 | 0 | 702 | 83 |
|  | 9 | 662 | 52 |
|  | 18 | 597 | 43 |
|  | 27 | 517 | 38 |
| 9 | 0 | 725 | 114 |
|  | 9 | 697 | 78 |
|  | 18 | 632 | 55 |
|  | 27 | 545 | 45 |
| 10 | 0 | 783 | 130 |
|  | 9 | 742 | 80 |
|  | 18 | 671 | 52 |
|  | 27 | 593 | 50 |
| 11 | 0 | 821 | 87 |
|  | 9 | 788 | 61 |
|  | 18 | 727 | 50 |
|  | 27 | 645 | 52 |
